# Antibiofilm and antibacterial effects of specific chitosan molecules on *Staphylococcus aureus* isolates associated with bovine mastitis

**DOI:** 10.1371/journal.pone.0176988

**Published:** 2017-05-09

**Authors:** Abdelhamid Asli, Eric Brouillette, Céline Ster, Mariana Gabriela Ghinet, Ryszard Brzezinski, Pierre Lacasse, Mario Jacques, François Malouin

**Affiliations:** 1 Centre d’Étude et de Valorisation de la Diversité Microbienne (CEVDM), Département de biologie, Faculté des sciences, Université de Sherbrooke, Sherbrooke, Québec, Canada; 2 Canadian Bovine Mastitis and Milk Quality Research Network (CBMMQRN) and *Regroupement de recherche pour un lait de qualité optimale* (Op+Lait), Université de Montréal; 3 Sherbrooke Research and Development Centre, Agriculture and Agri-Food Canada, Sherbrooke, Québec, Canada; 4 Département de pathologie et microbiologie, Faculté de médecine vétérinaire, Université de Montréal, St-Hyacinthe, Québec, Canada; Universitatsklinikum Munster, GERMANY

## Abstract

*Staphylococcus aureus* is one of the major pathogens causing bovine intramammary infections (IMIs) and mastitis. Mastitis is the primary cause for the use of antibiotics in dairy farms but therapeutic failure is often observed. One of the reasons for the lack of effectiveness of antibiotic therapy despite the observed susceptibility of bacterial isolates *in vitro* are bacterial biofilms. In this study, we used chitosan of well-defined molecular weight (0.4–0.6, 1.3, 2.6 and 4.0 kDa) and investigated their antibiofilm and antibacterial activities in *in vitro* and *in vivo* models related to *S*. *aureus* IMIs. A chitosan of at least 6 units of glucosamine was necessary for maximum antibacterial activity. The 2.6 and 4.0 kDa forms were able to prevent biofilm production by the biofilm hyperproducer strain *S*. *aureus* 2117 and a bovine MRSA (methicillin-resistant *S*. *aureus*). The intramammary administration of the 2.6 kDa chitosan showed no adverse effects in mice or in cows, as opposed to the slight inflammatory effect observed in mammary glands with the 4.0 kDa derivative. The 2.6 kDa chitosan killed bacteria embedded in pre-established biofilms in a dose-dependent manner with a >3 log10 reduction in CFU at 4 mg/ml. Also, the 2.6 kDa chitosan could prevent the persistence of the internalized MRSA into the mammary epithelial cell line MAC-T. An *in vitro* checkerboard assay showed that the 2.6 kDa chitosan produced a synergy with the macrolide class of antibiotics (*e*.*g*., tilmicosin) and reduced the MIC of both molecules by 2–8 times. Finally, the intramammary administration of the 2.6 kDa chitosan alone (*P*<0.01) or in combination with tilmicosin (*P*<0.0001) reduced the colonization of mammary glands in a murine IMI model. Our results suggest that the use of chitosan alone or in combination with a low dose of a macrolide could help reduce antibiotic use in dairy farms.

## Introduction

Mastitis is a major disease for dairy cattle. Mastitis decreases milk quality and production and reduces profitability because of the cost of treatment and discarded milk due to the risk of antibiotic residues in milk [1]. An intramammary infection (IMI) often occurs when bacteria invade the mammary gland through the teat canal causing an inflammatory response, which translates in either subclinical (visual symptoms are absent) or clinical mastitis (local and/or systemic symptoms are visible) [2, 3]. The expression of bacterial virulence factors influences the severity of mammary gland inflammation. Many Gram-positive and Gram-negative pathogens can cause mastitis [4]. Most of the time, *S*. *aureus* causes subclinical and persistent IMIs that often remain undetected and poorly respond to antibiotic treatment [5, 6]. *S*. *aureus* is also frequently isolated from clinical cases [5]. The existence of such strains represents a reservoir that causes the recurrence of *S*. *aureus* infections in herds [7]. Prevalence values of 3.4 to 8.2% for quarter infections have been observed in several studies where milk samples have been taken randomly from quarters of all cows in herds [8, 9, 10].

*S*. *aureus* is able to produce a number of virulence factors that contribute in causing and maintaining an IMI [11]. These factors belong to different groups including surface-associated components and degradative enzymes as well as toxins such as the staphylococcal enterotoxins and toxic shock syndrome toxin-1 (TSST-1) [11]. Furthermore, most of *S*. *aureus* strains isolated from bovine mastitis are able to produce alpha- and beta-hemolysins and leukocidins provoking host cell destruction [12, 13]. Other molecules like phenol-soluble-modulins (PSM) play a major role in *S*. *aureus* pathogenesis. Indeed, PSM are peptides that facilitate lysis of white blood cells like neutrophils after phagocytosis [14]. *S*. *aureus* is responsible for causing several types of infections, many of which are related to biofilm production [15]. The biofilm is defined as a community of bacteria attached to biotic or abiotic surfaces and embedded in a self-synthesized organic polymer matrix [16]. The major component of the extracellular polymer matrix is the polysaccharide intercellular adhesin (PIA), whose synthesis is directed by the *icaADBC* operon [17]. The staphylococcal biofilm is likely a key virulence factor involved in the persistence of the bacteria during bovine mastitis [18]. Indeed, a previous study from our laboratory showed that some clones of *S*. *aureus* produce significantly more biofilm than others and that strains that are not eliminated during the antibiotic therapy of the dry period, produce more biofilm than strains that are eliminated after calving [19]. Furthermore, biofilms reduce the effectiveness of antimicrobial peptides, which are important constituents of the host innate immune system, reducing phagocytosis during infection and increasing resistance to antibiotics [15, 20]. A relationship between the *icaADBC* operon and the accessory gene regulator (*agr*) locus, a quorum-sensing system that regulates the expression of many virulence factors in *S*. *aureus*, has been elucidated. Some studies have reported that low *agr* activity is required to support biofilm formation through the up-regulation of bacterial surface components or adhesins whereas the dispersion of biofilm is controlled by the secretion of proteases and nucleases, which are stimulated by *agr* activation [21, 22]. Thus, biofilm production is a significant contributor to *S*. *aureus* pathogenesis and the need for alternative therapies that directly tackle this element is of utmost importance.

In the last decades, many natural compounds have been employed in an attempt to disrupt biofilm-associated infections. Such compounds were many but included eugenol [23], lectins [24] and chitosan [25]. In particular, chitosan has been investigated as an antimicrobial agent against a vast range of organisms such as algae, bacteria, yeasts and fungi in experiments involving *in vitro* and *in vivo* interactions with chitosan in different forms (films, solutions and composites) [26]. Chitosan is defined as a linear polysaccharide, derived from the naturally abundant chitin, and composed of D-glucosamine and N-acetyl-D-glucosamine units linked by β-1,4-glycosidic linkages [27]. The biological activity of chitosan is dependent on several factors that can considerably vary or that can be modified: the degree of polymerization (DP) or molecular weight (MW), and the degree of deacetylation (DD) are the main known factors [28]. The biodegradability, biocompatibility and non-toxicity, as well as hypoallergenic properties of chitosan have been reported, which justify the use of this polysaccharide in a wide range of possible applications in the pharmaceutical, biomedical, industrial and agricultural fields [29, 30, 31]. Some studies have reported the antibiofilm effect of low molecular weight chitosan (LMWC) hydrogel *in vitro* and *in vivo*. Indeed, LMWC hydrogel (MW 50 kDa, DD 92%) was shown to significantly inhibit biofilm formation by *Candida parapsilosis in vitro* and *in vivo* using a catheter mouse model [32]. Similarly, LMWC (MW 107 kDa, DD 75–85%) demonstrated a strong ability to inhibit biofilm production by *S*. *epidermidis* and *C*. *albicans in vitro* but was not equally efficient against *S*. *aureus* strains [33].

In this study, we evaluated the antibiofilm and antibacterial effects of chitosan on *S*. *aureus*. We hypothesized that polycationic polymers such as chitosan could be used to treat biofilm-associated infections caused by *S*. *aureus* isolates associated with bovine mastitis. Most importantly, the use of well-defined forms of chitosan should help segregating some of its important biological activities. In addition, we have explored the potential benefits of using chitosan in combination with low amounts of antibiotics in an effort to reduce antibiotic use in dairy farms. We show here that the combination of chitosan with antibiotics could be useful to enhance the killing of bacteria within preformed biofilms and in a murine model of *S*. *aureus* IMI.

## Materials and methods

### Bacterial strains

The isolates were selected from the Mastitis Pathogen Culture Collection (MPCC) belonging to the Canadian Bovine Mastitis and Milk Quality Research Network (CBMMQRN, St-Hyacinthe, QC, Canada). They include one bovine MRSA (strain 1158c, *spa* type t451, barcode 10812464) and one biofilm hyperproducer (strain 2117, *spa* type t13401, barcode 10705001), evidenced in a previous study [19]. The reference strain for antibiotic susceptibility tests (*S*. *aureus* ATCC 29213) and the bovine strain Newbould (ATCC 29740) were obtained from the American Type Culture Collection (ATCC, Manassas, Virginia).

### Chitosan molecules

Chitosan molecules with a DP of ~2 to 4 oligosaccharide units (CHOS, with a number average molecular weight [Mn] of 0.4–0.6 kDa), and oligosaccharides of low molecular weight, Mn of 1.3 kDa (DP ~6 units), Mn of 2.6 kDa (DP ~13 units), and Mn of 4 kDa (DP ~20 units) were prepared from 98% N-deacetylated chitosan (Marinard Biotech, Québec, Canada) by total or partial hydrolysis with endo-chitosanase from *Streptomyces sp*. N174 [34]. Chitosan (25 g/L) was dissolved in 0.2 M acetic acid, mixed up with the chitosanase (0.1 Unit per gram of chitosan), and incubated for several hours depending of the weight desired at 37°C. The reaction was stopped by heating for 5 min in boiling water. The Mn was estimated by the reducing sugar assay [35]. The hydrolyzed chitosan was recovered by lyophilization. The lyophilized product contained around 1% of residual acetate.

### Minimal inhibitory concentration (MIC)

The MICs were determined by using a broth microdilution technique in 96-well plates following the recommendations of the Clinical and Laboratory Standards Institute [36]. Chitosan molecules were prepared as described above and traditional antibiotics were purchased from Sigma Aldrich (Oakville, ON, Canada). Each compound was serially diluted 2-fold with cation-adjusted Mueller-Hinton broth (CAMHB) in 96-well plates. Bacteria were inoculated at 10^5^ CFU/ml and plates were incubated at 35°C for 18–24 h. Bacterial growth was quantified by measuring the optical density at 600 nm (OD 600 nm) using a micro plate reader (Epoch microplate spectrophotometer, Bio-Tek Instruments, Winooski, VT). The MIC value represented the lowest concentration of compound that has an OD 600 nm identical to the control well containing only CAMHB (*i*.*e*., no visible growth).

### Synergy test

A 96-well plate checkerboard assay was used to identify possible synergy between chitosan and antibiotics. The first antibiotic of the combination was serially diluted 2-fold with CAMHB along the ordinate, while the second drug was diluted along the abscissa [37]. All the wells were inoculated with 10^5^ CFU/ml prepared in CAMHB and the plates were incubated at 35°C for 24 h. The resulting checkerboard contains each combination of two antibiotics. The ∑FICs (fractional inhibitory concentration index) were calculated as follows: ∑FIC = FIC A + FIC B, where FIC A is the MIC of drug A in the combination/MIC of drug A alone, and FIC B is the MIC of drug B in the combination/MIC of drug B alone. The combination considered synergistic when the ∑FIC is ≤0.5, indifferent or additive when the 0.5< ∑FIC <2, and antagonistic when the ∑FIC is ≥2 [38].

### Kill kinetics

Kill kinetics experiments were performed in order to evaluate the bactericidal effect of chitosan. Bacteria were inoculated at 10^5^ CFU/ml in CAMHB in the presence or absence of chitosan. At several points of time, bacteria were sampled, serially diluted and plated on tryptic soy agar (TSA) plates for CFU counts. Plates were incubated for 24 h at 35°C. The detection limit was 10 CFU/ml.

### Biofilm production assay

Biofilm production was assessed in sterile flat-bottom 96-well polystyrene microtiter plates. The bacterial isolates were inoculated into a brain heart infusion (BHI) supplemented with 0.25% glucose (BHIg) and grown for 18 to 24 h at 35°C. A cell suspension adjusted to a 0.5 McFarland standard was prepared for each bacterial strain and a volume of 100 μl was added to the wells containing different forms of chitosan serially diluted 2-fold with BHIg to obtain a final volume of 200 μL per well. The plates were then incubated at 35°C without agitation for 24h. After incubation, the growth was evaluated by measuring the OD at 600 nm with an Epoch microplate spectrophotometer before discarding the medium. The wells were gently washed three times with 200 μL of sterile phosphate buffered saline, pH 7.4 (PBS), air dried, and stained with 200 μL of 0.01% crystal violet for 30 min. Each well was re-washed three times with 200 μL of sterile distilled water prior to the addition of 200 μL of 95% ethanol to dissolve the biofilm stain. Finally, the biofilm produced in each well was measured respective to the OD at 540 nm and normalized to the growth (OD 600 nm). Measurements were done in quadruplicate, and each assay was repeated three times.

To measure the ability of chitosan molecules to disrupt preformed biofilms, bacteria were grown for 24 h in 96-well plates with lids having pegs (Thermo Scientific, Ottawa, ON) according to the Biofilm Calgary Device method [39]. The biofilm formed on pegs were washed three times with 200 μL PBS and were further incubated in new 96-well plates carrying 200 μL of fresh BHIg containing serial dilutions of tested compounds. The plates were then incubated for another 24h at 35°C, 120 RPM. The treated biofilm on pegs were washed three times with PBS, air dried, and stained with 200 μL of 0.1% crystal violet for 30 min. All the pegs were washed again twice with 200 μL of sterile distilled water prior to addition of 200 μL of 95% ethanol, and the OD was measured at 540 nm. Measurements were done in triplicate, and each experiment was repeated three times.

### Bactericidal activity of chitosan alone or in combination with an antibiotic in preformed biofilms

The viability of bacteria in preformed biofilms treated with chitosan alone or in combination with an antibiotic was evaluated using the peg lid method as described above with the following modifications. The biofilms formed on pegs were washed three times with 200 μL of PBS and were further incubated in new 96-well plates carrying 200 μL of fresh BHIg containing serial dilutions of test compounds (chitosans, antibiotics or a combination). The plates were then incubated for another 24 h at 35°C, 120 RPM. The treated biofilm on pegs were washed three times with PBS and the bacteria were recovered by sonication in a new 96-well plate containing 200 μL of PBS per well using an ultra-sonicator bath for 10 min. To recover all the detached bacteria, the 96-well plate was centrifuged for 5 min at 1000 RPM. A volume of 190 μL was recovered from each well, serially diluted, plated on TSA and incubated at 35°C for 24 h before CFU counting. Measurements were done in triplicate, and each experiment was repeated at least twice.

### Cytotoxicity assay

MAC-T bovine mammary epithelial cells were used to evaluate the relative cytotoxicity of chitosan by measuring the release of lactate dehydrogenase (LDH) from cells. Briefly, MAC-T cells were seeded in 48-well plates using complete medium (DMEM, Dulbecco's Modified Eagle's Medium; Sigma Aldrich, Oakville, ON, Canada), 10% of fetal bovine serum, antibiotic antimitotic solution (100 IU/ml of Penicillin sodium salt, 100 μg/ml of Streptomycin sulfate and 0.25 μg/ml of Amphotericin B), sodium pyruvate (1 mM), insulin (5 μg/ml) and hydrocortisone (1 μg/ml)) for 30% of confluence in order to reach 100% of confluence after 2 days of incubation at 37°C with 5% CO_2_. After incubation, the cells were washed twice with 1 mL of DMEM pre-warmed at 37°C prior to the addition of 200 μl of a pre-warmed invasion medium (DMEM containing 1% of fetal bovine serum, sodium pyruvate (1 mM), insulin (5 μg/ml) and hydrocortisone (1 μg/ml)). A volume of 50 μl of chitosan (5 × concentrated), 50 μl of the invasion medium acting as the low-toxicity control and 50 μl of Triton x-100 (5% v/v) as the high-toxicity control were added to the respective wells. After 6 h of incubation at 37°C with 5% CO_2_, a volume of 200 μl was recovered from the wells and centrifuged at 250 *g* in order to remove the cells from the culture medium. In 96-well plate, 100 μl of the recovered supernatant were mixed with 100 μl of the reaction mixture from the LDH detection kit (Roche Diagnosis, Indianapolis, IN) and the plate was incubated for 15 min at room temperature prior to measuring the OD at 492 nm and 655 nm using an Epoch microplate spectrophotometer, according to the enclosed protocol provided by the manufacturer.

### Effect of chitosan on the internalization and persistence of bacteria into MAC-T cells

MAC-T bovine mammary epithelial cells were used to evaluate the ability of chitosan to prevent the internalization and persistence of bacteria within cells. Briefly, MAC-T cells were seeded in 24-well plates using the DMEM complete medium for a confluence of 30% in order to obtain 100% of confluence after 2 days of incubation at 37°C with 5% CO_2_. MAC-T cells were washed with pre-warmed DMEM prior to the addition of the invasion medium. The day of the experiment, bacteria from an overnight culture were diluted 20 times and grown in a fresh tryptic soy broth for 2 h at 37°C. Bacteria were centrifuged at 3000 RPM for 10 min and washed twice with cold sterile PBS. Bacteria in PBS at ~4 ×10^6^ CFU/ml (multiplicity of infection [MOI] of 10, *i*.*e*., the ratio bacteria/cells) were mixed with chitosan at different concentrations prior to addition to the wells containing confluent MAC-T cells. After 3 h of invasion, the cells were washed with DMEM prior to the addition of invasion medium containing lysostaphin (20 μg/ml) and incubated for 30 min (or for 24 h for the intracellular persistence test) at 37°C with 5% CO_2_. After incubation, infected MAC-T cells were washed with DMEM and treated with trypsin 0.25% for 10 min at 37°C prior to the addition of sterile water to lyse the MAC-T cells and release the intracellular bacteria. Immediately after, PBS (10 ×) was added and CFU counts were obtained after plating serial dilutions of the cell lysate on TSA.

### Safety assessment in animals

The innocuity of the different forms of chitosan was evaluated *in vivo* using a mouse mastitis model and cows. Briefly, CD-1 lactating mice were separated from their pups, anesthetized, and received two different doses of chitosan (0.1 mg/gland or 2.5 mg/gland). First, the fourth pair of glands found from the head to tail (L4 and R4 glands) was disinfected with 70% ethanol. Then, 50 μL of chitosan (in PBS) was slowly injected into the lactiferous duct with a 32-gauge blunt needle attached to a 1 ml syringe. Fourteen hours later, mammary glands were harvested and a visual observation performed on tissue. Inflammation scores were given depending on the mammary gland redness.

In order to evaluate the innocuity of the different forms of chitosan in cows, each of three mammary gland quarters received either 500 mg of chitosan (2.6 kDa or 4 kDa) or saline as the negative control after the morning milking. Milk samples were collected aseptically from each quarter before chitosan treatment as well as 2, 4, 8, 21, 33, 45 and 69 h after intra-mammary instillation of chitosan to evaluate the onset of inflammation by determining the somatic cell count (SCC). Cows were milked every 12 h using a quarter-milking machine in order to get individual quarter milk production.

### Murine mastitis model

A mouse mastitis model [40, 41] was used to evaluate the capacity of chitosan alone or in combination with an antibiotic to reduce *S*. *aureus* colonization of mouse mammary glands. Briefly, CD-1 lactating mice were separated from their pups, anesthetized with ketamine and xylazine at 87 and 13 mg/kg of body weight, respectively, and mammary glands were infected with *S*. *aureus* Newbould or *S*. *aureus* 2117 (biofilm hyperproducing strain). First, the fourth pair of glands found from the head to tail (L4 and R4 glands) was disinfected with 70% ethanol. Then, 100 μl of PBS containing ~100 bacterial CFUs was slowly injected into the lactiferous duct with a 32-gauge blunt needle attached to a 1 ml syringe. Two doses of chitosan (in PBS) were administered into the infected mammary glands: one dose was administered immediately after inoculation of bacteria and the second dose was given four hours post-inoculation alone or in combination with tilmicosin, after anesthetizing mice. Twelve hours later, *i*.*e*. 16 h after bacterial inoculation, mammary glands were harvested and homogenized. CFU counts were obtained after plating serial dilution of mammary gland homogenates on TSA. The detection limit was approximately 200 CFU per gram of mammary gland.

### Statistical analysis

Most statistical analyses were carried out with the GraphPad Prism software (v.6.02). Bacterial CFUs were transformed in base 10 logarithm values before being used for statistical analyses. Statistical tests used for the analysis of each experiment and significance are specified in the figure legends. For experiments performed with cows, data were analyzed by ANOVA using the MIXED procedure of SAS (SAS Institute Inc., Cary, NC) and details are also specified in the figure legends.

### Ethics statement

All the experiments performed with animals were approved by the ethics committee on animal experimentation of the Faculté des sciences of the Université de Sherbrooke (mice) and by the Agriculture and Agri-Food Canada local institutional animal care committee (cows), and were conducted in accordance with the guidelines of the Canadian Council on Animal Care.

## Results

### Comparison of chitosan forms based on *in vitro* antibacterial and antibiofilm activities

Bacterial susceptibility tests were performed for a variety of chitosan forms having different degrees of polymerization (DP). The different forms of chitosan used in this assay were CHOS (0.4–0.6 kDa), 1.3 kDa, 2.6 kDa and 4.0 kDa. The MICs obtained with CHOS were 16 mg/ml whereas the 1.3 kDa, 2.6 kDa and 4.0 kDa exhibited much lower MICs (1 mg/ml) for all strains tested (Table 1). The different forms of chitosan were highly deacetylated (98%) and thus the unique difference was the DP. The results show that an oligosaccharide of at least 6 units is necessary for maximum antibacterial activity.

**Table 1:** MIC values of the various forms of chitosan against *S*. *aureus* strains.

		MIC (mg/ml)			
		CHOS	1.3 kDa	2.6 kDa	4.0 kDa
		DP 2–3	DP 6	DP 13	DP 20
	Strain				
*S*. *aureus* ATCC 29213		16	1	1	1
*S*. *aureus* 2117		16	1	1	1
MRSA 1158c		16	1	1	1

DP: Degree of polymerization

**Fig 1:**
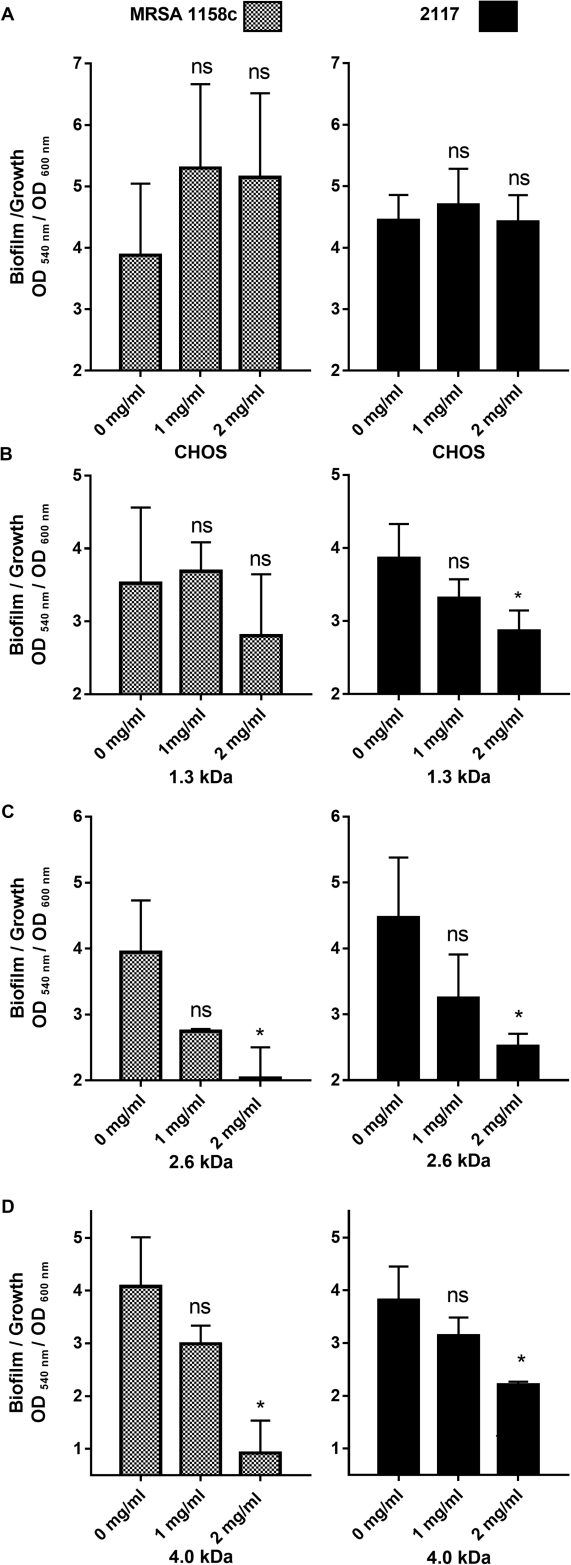
Antibiofilm activity of the different forms of chitosan against MRSA 1158c and the biofilm hyperproducer strain 2117. (A) CHOS, (B) 1.3 kDa, (C) 2.6 kDa, and (D) 4.0 kDa chitosan. Bars represent the means and vertical lines the standard deviation (SD). Data were obtained from three independent experiments. Significant differences in comparison to the untreated control (0 mg/ml) are shown by asterisks. Statistical analysis was performed using Kruskal-Wallis test (non-parametric one way ANOVA) with Dunn’s multiple comparison test: ns, non significant; *, *P*<0.05.

In order to delineate the antibiofilm activity of the different forms of chitosan, we first measured their effects on biofilm production by *S*. *aureus* in 96-well plates. Fig 1 shows the effect of different forms of chitosan on biofilm formation by the bovine MRSA strain and the biofilm hyperproducer strain 2117. To distinguish the antibiofilm activity from a possible growth inhibitory effect by chitosan, we used the OD ratio 540/600 nm, which relates biofilm production to the overall growth. Using such a ratio, we found that CHOS was only able to inhibit *S*. *aureus* biofilm production using a concentration as high as 16 mg/ml (data not shown), which is at or near its MIC. Note that MICs are determined using an inoculum of 10^5^−10^6^ CFU/ml, whereas in this biofilm assay the inoculum was 10^8^ CFU/ml, a condition that allows visible growth at concentrations up to 4 × MIC. As for the other forms of chitosan, a concentration of 2 mg/ml caused a significant decrease in the biofilm/growth ratio except for the 1.3 kDa form, which only significantly affected strain *S*. *aureus* bovine 2117.

Together with the MIC results, it seems that the 2.6 and the 4.0 kDa forms had the best antibacterial and antibiofilm activities against *S*. *aureus*. Those two forms were therefore further evaluated and compared based on cytotoxicity and safety issues, considering an eventual use of such molecules for the treatment of bovine mastitis.

### Cytotoxicity and safety assessment in animals

First, the innocuity of the different forms of chitosan was evaluated using two different doses (0.1 mg and 2.5 mg) administered by direct intramammary injection in mice. The results are presented in Table 2. CHOS and the 2.6 kDa chitosan demonstrated a weak or null score of inflammation, compared to the 4.0 kDa molecule showing a score of 2.75 (a score of 3 being the maximum) at the highest dose administered (2.5 mg/gland).

**Table 2:** Inflammation scores obtained after administration of chitosan by intramammary injection in mice.

	Inflammation scores ^a^	
Chitosan forms	Dose administered per gland (mg)	
	0.1	2.5
CHOS	0 ± 0	0.17 ± 0.29
2.6 kDa	0.17 ± 0.29	0 ± 0
4.0 kDa	0 ± 0	2.75 ± 0.35

^a^ Inflammation scores were given depending on the mammary gland shape. The inflammation can be: 0: none or slight, 1: moderate, 2: medium and 3: severe. The values represent the mean of three observations of three different glands (3 mice) and standard deviations.

**Fig 2:**
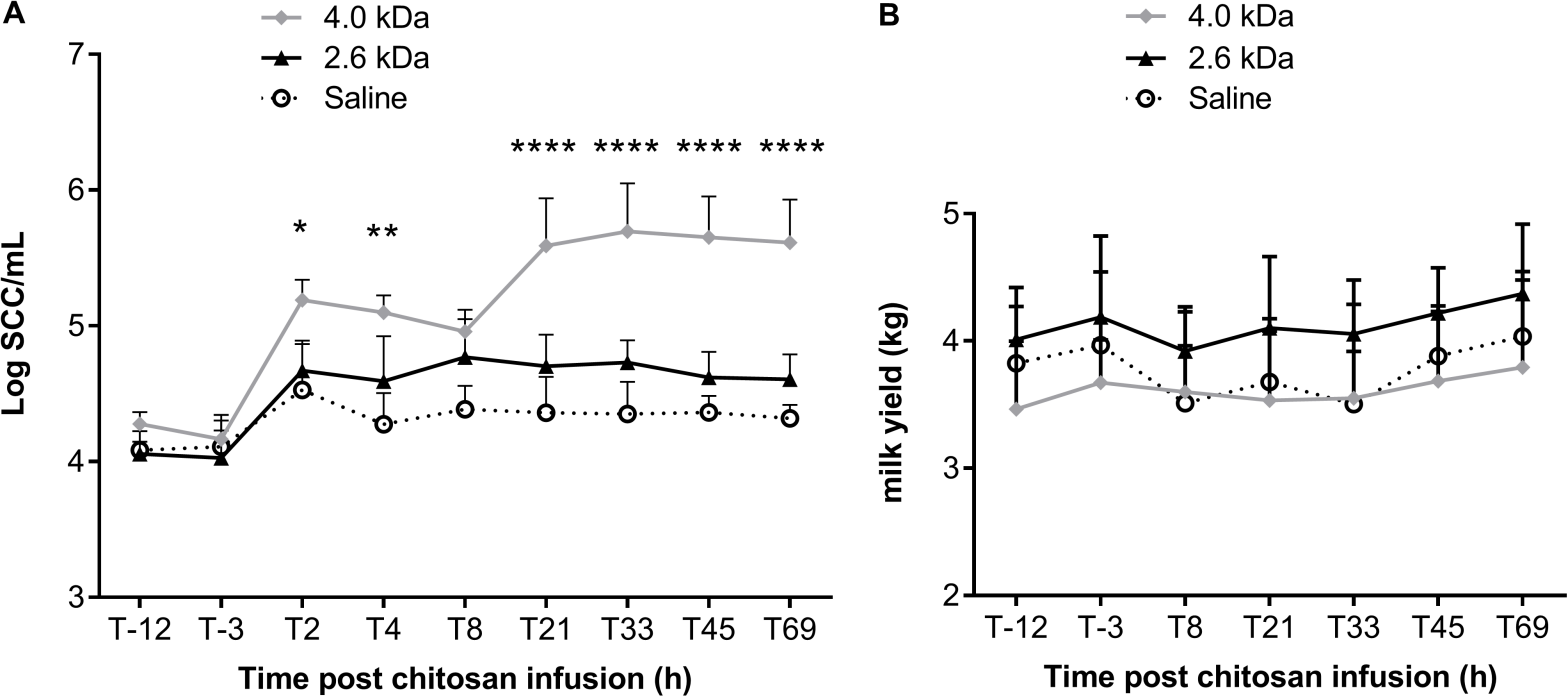
Relative innocuity of different forms of chitosan in cows. Each quarter of cow’s udder has received 500 mg of chitosan (2.6 kDa or 4.0 kDa) or saline as the negative control. Milk samples and somatic cell counts (SCC) were determined 12 and 3 hours before the instillation of chitosan. After the intramammary instillation, milk samples were aseptically collected from cows at several points in time to evaluate inflammation by determining the SCC (A) and milk yields (B). Symbols represent the means and vertical lines the standard deviation. Data were analyzed by ANOVA using the MIXED procedure of SAS (SAS Institute Inc., Cary, NC). For the first experiment, time was used as a repeated effect and treatment (cow) was used as the subject. Orthogonal contrasts were performed to compare the effect of each treatment to control. No difference was observed between saline and the 2.6 kDa chitosan (SCC and quarter milk yield). Significant differences were observed between saline and 4.0 kDa for SCC: *, *P*<0.05; **, *P*< 0.01; ***, *P*<0.0001.

In cows, there was a Treatment × Time interaction (*P*<0.001), meaning that the effect of treatment depends on time. Somatic cell counts (SCC) in milk of quarters treated with the 4.0 kDa chitosan were significantly higher than those measured for quarters treated with saline during the whole monitoring period (*P*<0.001). The intramammary injection of the 2.6 kDa chitosan tended to slightly increase SCC between 4 h and 33 h post injection but this difference was not statistically significant (*P*<0.1, Fig 2A). Quarter milk yield was not affected by treatments (Fig 2B).

**Fig 3:**
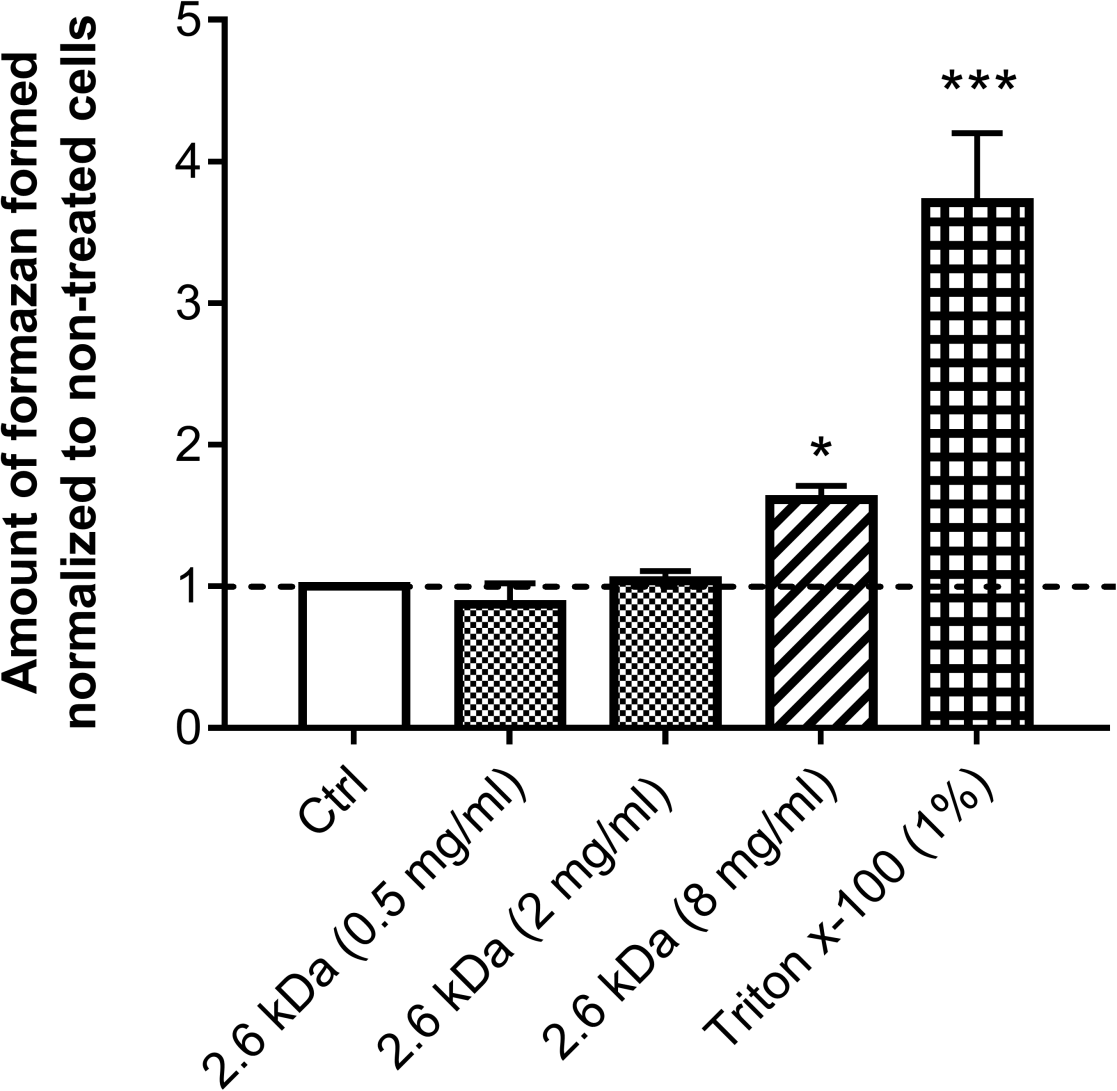
Effect of the 2.6 kDa chitosan on LDH release from MAC-T bovine mammary epithelial cells. Cells were exposed to the 2.6 kDa chitosan at different concentrations (0.5 mg/ml, 2 mg/ml and 8 mg/ml). Triton x-100 and culture medium were used as cytotoxic positive and negative (Ctrl) controls, respectively. The amount of formazan formed was normalized to the amount formed by the non-treated cells (Ctrl). Bars represent the means and vertical lines the standard deviation (SD). Significant differences in comparison to the negative control (Ctrl) are shown. Statistical analysis was performed using the Kruskal-Wallis test (non-parametric one way ANOVA) with Dunn’s multiple comparison test: *, *P*<0.05; ***, *P*<0.0001.

Because the 2.6 kDa molecule appeared to be the safest candidate, MAC-T bovine mammary epithelial cells were then used to further evaluate its cytotoxicity by assessing LDH release resulting from cell membrane damage (Fig 3). No cell toxicity was observed when the 2.6 kDa molecule was used at 0.5 mg/ml and 2 mg/ml (0.5 × and 2 ×MIC, respectively). A slight cytotoxicity was observed compared to the control when the 2.6 kDa chitosan was used at 8 mg/ml (8 × MIC).

This initial safety assessment of chitosan showed that the 2.6 kDa molecule has a promising safety profile and this warranted further investigation of its antibiotic and antibiofilm properties (below).

### Bactericidal activity of the 2.6 kDa chitosan and synergy with antibiotics

**Fig 4:**
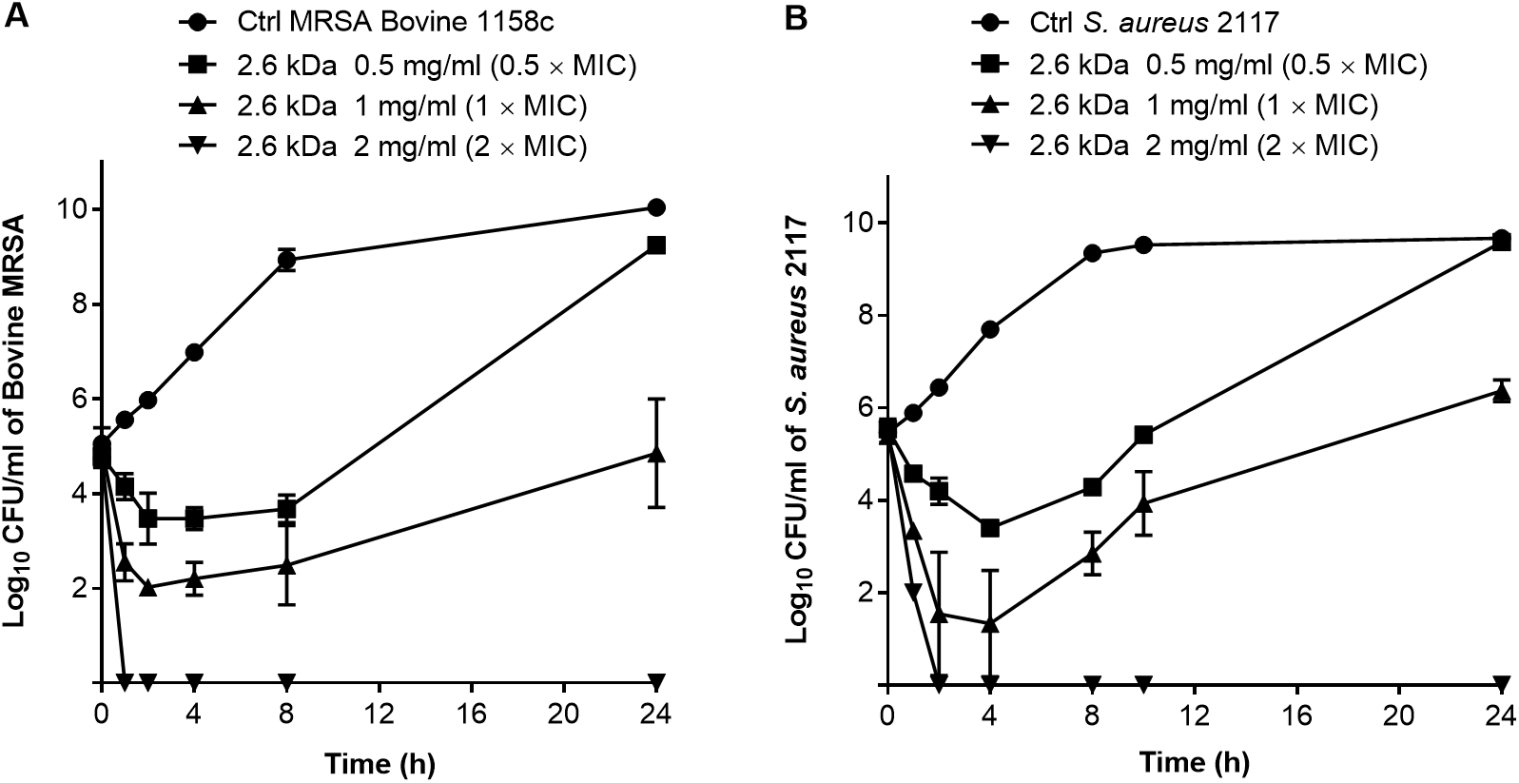
**Time-kill experiments showing the viability of *S*. *aureus* untreated (Ctrl) or in the presence of increasing concentrations of the 2.6 kDa chitosan, (A) for strain MRSA 1158c, and (B) for the biofilm hyperproducer strain 2117.** The CFU detection limit is 10 CFU/ml. Data are presented as means with standard deviations from three independent experiments.

The bactericidal activity of the 2.6 kDa chitosan was determined in time-kill experiments. Fig 4 shows a strong dose-dependent bactericidal activity of this form of chitosan used at 2 × MIC (*i*.*e*., 2 mg/ml) against both the bovine MRSA and the biofilm hyperproducer strains (>3 log10 drop in CFU/ml compared to the initial inoculum). Note that the pH of medium with the highest concentration of chitosan (2 mg/ml) was 5.94 as compared to 6.60 for the CAMHB medium without supplementation. Such a slight pH variation cannot explain the kill kinetics observed in Fig 4.

**Fig 5:**
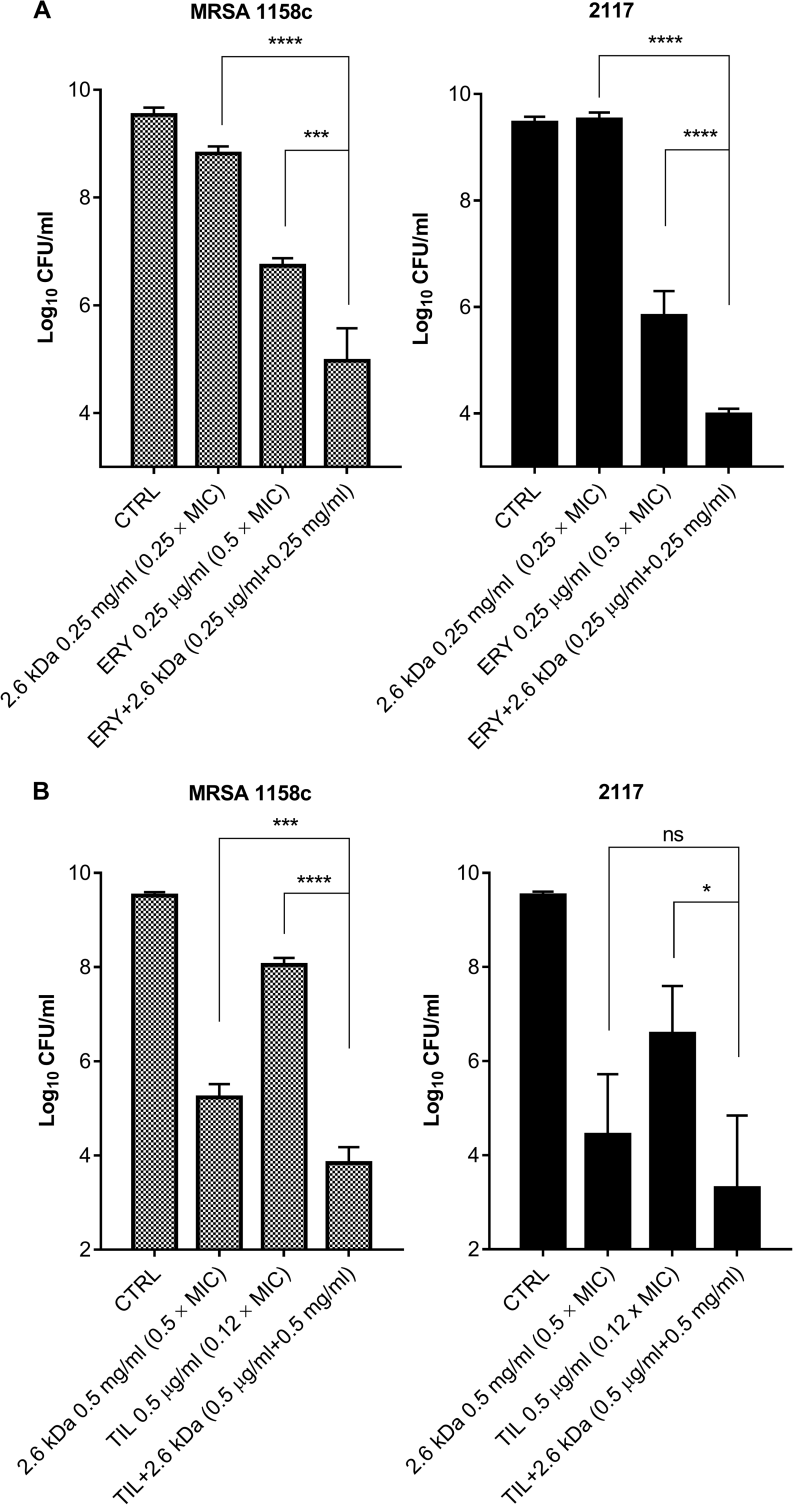
**Viability of *S*. *aureus* 2117 and MRSA 1158c either untreated (CTRL) or in the presence of a sub-MIC of chitosan (2.6 kDa) used alone or in combination with a sub-MIC of (A) erythromycin (ERY) or (B) tilmicosin (TIL) at 10 h post-treatment.** The sub-MIC concentrations are indicated on the graphs. The CFU detection limit is 10 CFU/ml. Data are presented as means with standard deviations from three independent experiments. Significant differences between the control (CTRL) and test conditions are shown (ns, non significant; *, *P*<0.01; ***, *P*<0.001; ****, *P*<0.0001 as determined by using one way ANOVA with Tukey’s multiple comparison test.

To build up on the observed bactericidal activity of chitosan, a checkerboard assay was then performed to detect any possible synergy between the 2.6 kDa chitosan and any of several classes of antibiotics against *S*. *aureus* ATCC 29213. The checkerboard result (Table 3) showed that the 2.6 kDa molecule exhibits synergy with erythromycin belonging to the macrolide class (∑ FIC value: 0.49) as well as with ciprofloxacin, a fluoroquinolone, (∑ FIC: 0.54) although the latter FIC was near the cut-off value (0.5). There were only indifferent or additive effects when chitosan was combined with the other classes of antibiotics. No antagonistic effect was observed. The 2.6 kDa chitosan also demonstrated synergy when combined with erythromycin against the bovine MRSA strain 1158c (∑ FIC: 0.49). Thus, sub-MICs of chitosan and erythromycin were combined to evaluate the bactericidal activity of such a drug combination in time-kill experiments. Fig 5A represents residual bacterial counts (CFU/ml) at 10 h post-treatment for both *S*. *aureus* 2117 and MRSA in presence of chitosan alone or in presence of erythromycin (both used at sub-MICs). Tilmicosin (MIC of 4 μg/ml), a macrolide analog of erythromycin usually used for treatment of bovine infections, was also used in a similar experiment (Fig 5B). In both cases, chitosan combined with the macrolide significantly reduced the bacterial counts compared to the use of the macrolide and/or chitosan alone, even when these compounds were used at sub-MICs.

**Table 3:** MIC values of antibiotics and chitosan (2.6 kDa) in a checkerboard assay against *S*. *aureus* ATCC 29213.

Antibiotics (ATB) in the checkerboard assay	ATB MIC alone (μg/ml)	ATB MIC with chitosan (μg/ml)	Chitosan MIC alone (mg/ml)	Chitosan MIC with ATB (mg/ml)	∑FIC value	Result^a^
Gentamicin	0.5	0.06	1	0.5	0.62	I
Erythromycin	0.5	0.12	1	0.25	0.49	S
Vancomycin	1	0.5	1	0.5	1.00	I
Chloramphenicol	4	2	1	0.5	1.00	I
Ciprofloxacin	0.25	0.01	1	0.5	0.54	BS^b^
Ceftiofur	0.5	0.5	1	0.5	1.50	I
Rifampicin	0.0025	0.0012	1	0.25	0.73	I
Pirlimycin	0.25	0.06	1	0.5	0.74	I
Oxacillin	0.12	0.12	1	0.5	1.50	I

^a^ I, indifferent, S, synergy.

^b^ borderline synergy.

### The 2.6 kDa chitosan kills bacteria embedded in preformed biofilms

**Fig 6:**
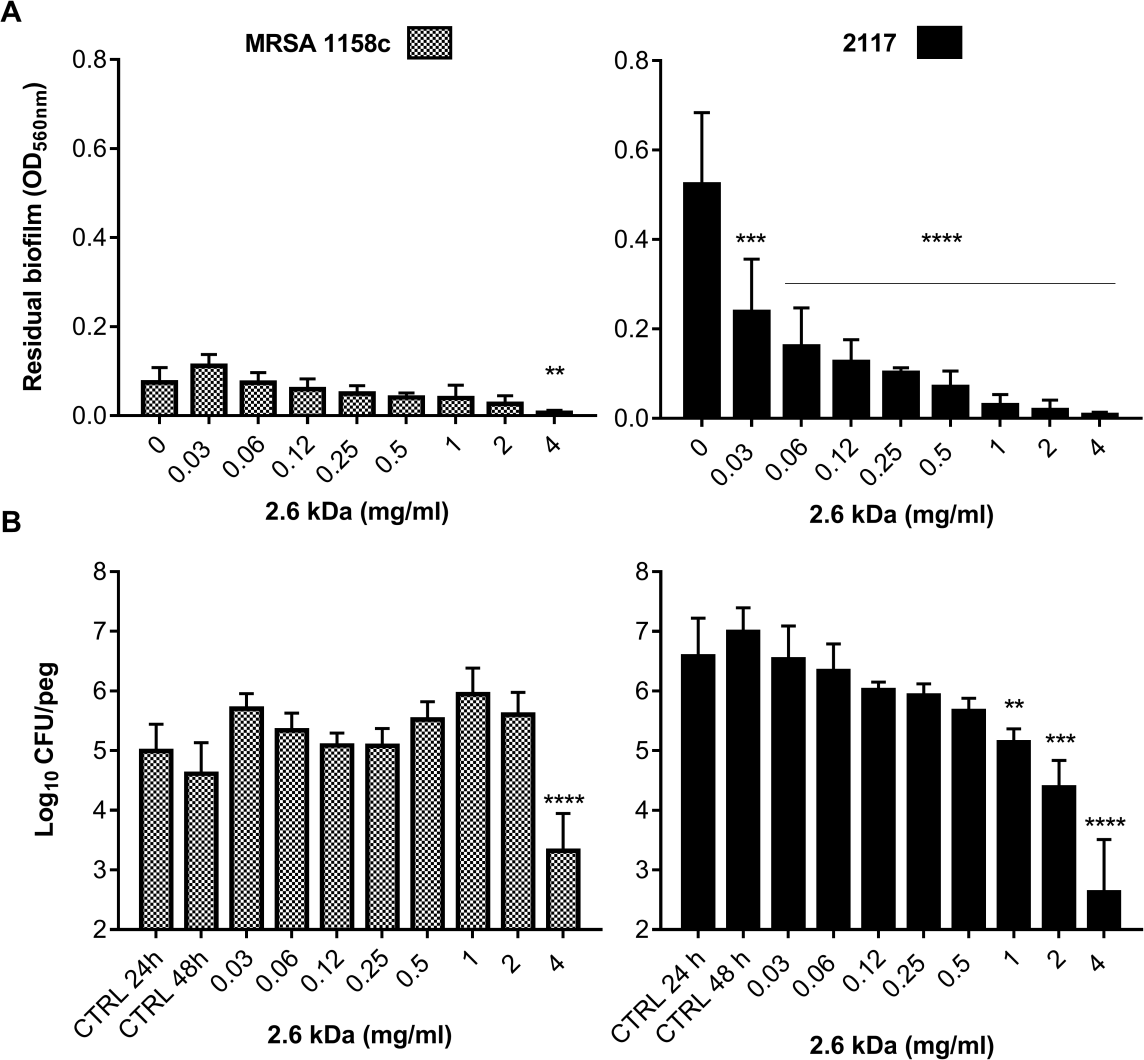
Preformed biofilms of *S*. *aureus* strains exposed to the 2.6 kDa chitosan. (A) Reduction of a preformed biofilm on pegs following exposure to increasing concentrations of chitosan. (B) Bactericidal effect of chitosan on preformed biofilms. The CFU/peg after 24 h of biofilm formation was evaluated for control pegs (CTRL 24 h) and this represented the inoculum at the onset of treatment, which occurred at 24 h for another 24 h of incubation. The CFU/peg obtained for the untreated pegs after the total incubation period served as the reference (CTRL 48 h) for treatment efficacy. Data were obtained from three independent experiments. Significant differences in comparison to the untreated controls (0 mg/ml in A, and CTRL 48 h in B) are shown. Statistical analysis was performed using non-parametric one way ANOVA: **, *P*<0.005; ***, *P*<0.001; ****, *P*<0.0001.

The Calgary biofilm device (96-well plate lids with pegs) was used to further evaluate the ability of the 2.6 kDa chitosan to disrupt a preformed biofilm of *S*. *aureus* 2117 and MRSA 1158c (Fig 6). Results show that the 2.6 kDa chitosan exhibited a dose-dependent activity against the preformed biofilm of MRSA 1158c (Fig 6A), which was significantly reduced by using a concentration of 4 mg/ml (4 × MIC). The preformed biofilm of the biofilm-hyperproducer strain 2117 was also significantly reduced in the presence of chitosan but distinctively, such an effect was observed at concentrations well below the MIC of chitosan (*i*.*e*., <1 mg/ml) with a strong and significant effect already observed at the lowest concentration tested (0.03 mg/ml).

Also, Fig 6B shows that the 2.6 kDa chitosan exhibited bactericidal activity against both *S*. *aureus* strains (MRSA 1158c and the biofilm hyperproducer strain 2117) pre-embedded in their biofilms. To some extent, these results paralleled those shown in Fig 6A. The use of 4 mg/ml (4 × MIC) of chitosan caused a 3.9 log10 and 1.6 log10 reduction in CFUs/peg for *S*. *aureus* 2117 and the MRSA strain, respectively, in comparison to the CFUs/peg of the untreated biofilm control at 48 h (Fig 6B). Interestingly, chitosan demonstrated a dose-dependent bactericidal activity on *S*. *aureus* 2117 and such an effect was significant at concentrations ≥ 1 mg/ml (Fig 6B). The latter observation paralleled the dose-dependent effect observed on the biofilm of that strain in Fig 6A.

### Bactericidal activity of a chitosan-tilmicosin combination against bacteria embedded in preformed biofilms

**Fig 7:**
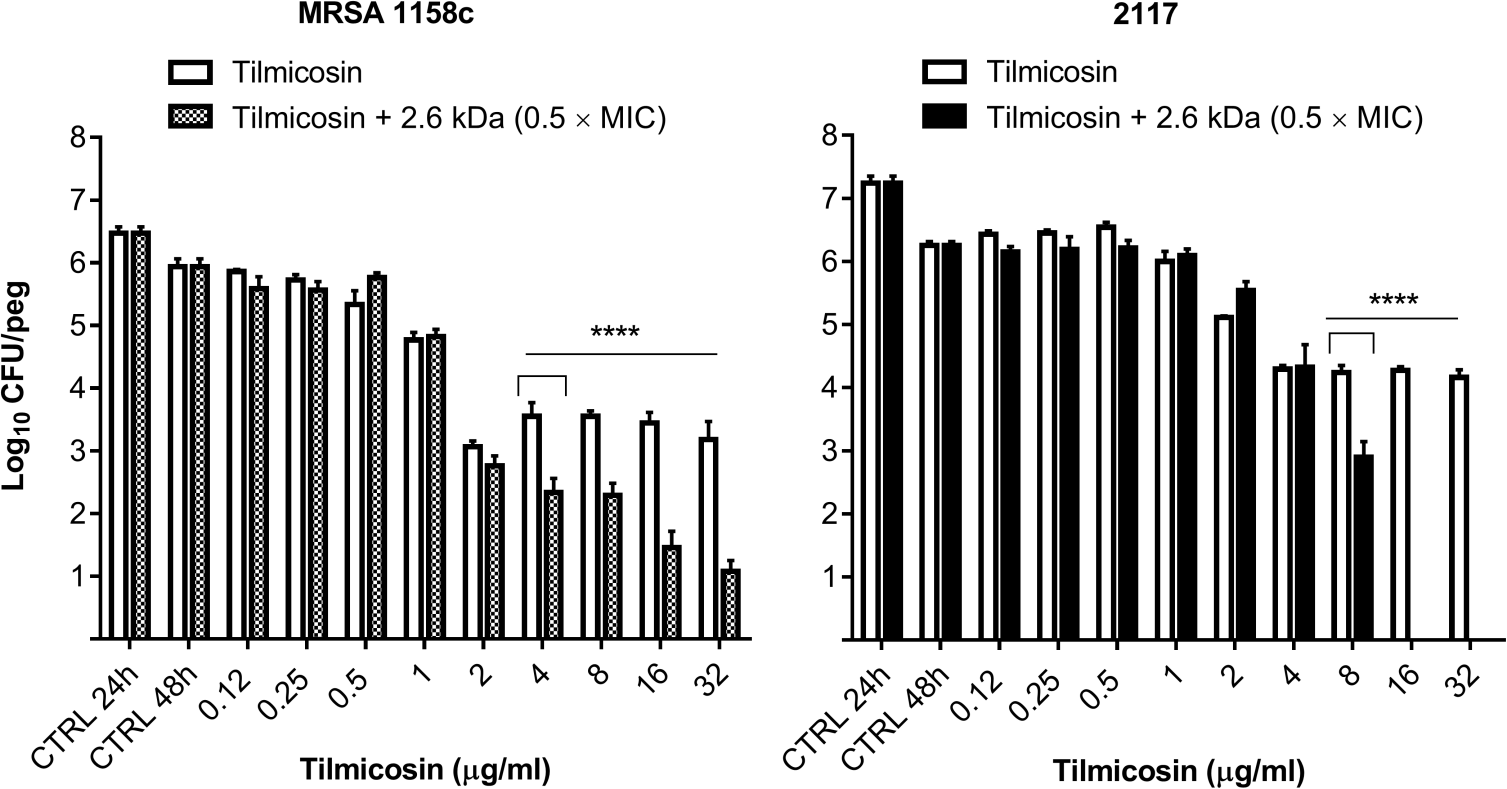
Bactericidal activity of increasing concentrations of tilmicosin used alone or in combination with a fixed sub-MIC of the 2.6 kDa chitosan against *S*. *aureus* strains embedded in preformed biofilms. The CTRL 24 h and CTRL 48 h are as defined in the legend of Fig 6. Data were obtained from three independent experiments. Significant differences between tests done with or without chitosan at each tilmicosin concentration are shown. Statistical analysis was performed using multiple t tests: ****, *P*<0.0001.

Next, based on data collected from biofilm assays, kill kinetics and observations of antibacterial synergy, it was appropriate to evaluate the bactericidal activity of tilmicosin alone or in combination with a fixed sub-MIC of the 2.6 kDa chitosan against bacteria embedded in preformed biofilms on pegs (Fig 7). Macrolide antibiotics are primarily bacteriostatic and it was therefore not surprising that tilmicosin used alone in this assay showed limited killing despite utilization of concentrations well above its MIC of 4 μg/ml against both *S*. *aureus* strains (Fig 7, white bars). In contrast, tilmicosin combined with the 2.6 kDa chitosan exhibited a dose-dependent activity against both *S*. *aureus* strains (Fig 7, grey or black bars). Thus, the addition of 0.5 mg/ml (0.5 × MIC) of chitosan enhanced the bactericidal activity of tilmicosin in biofilms.

### Effect of the 2.6 kDa chitosan on *S*. *aureus* internalization and persistence into MAC-T cells

**Fig 8:**
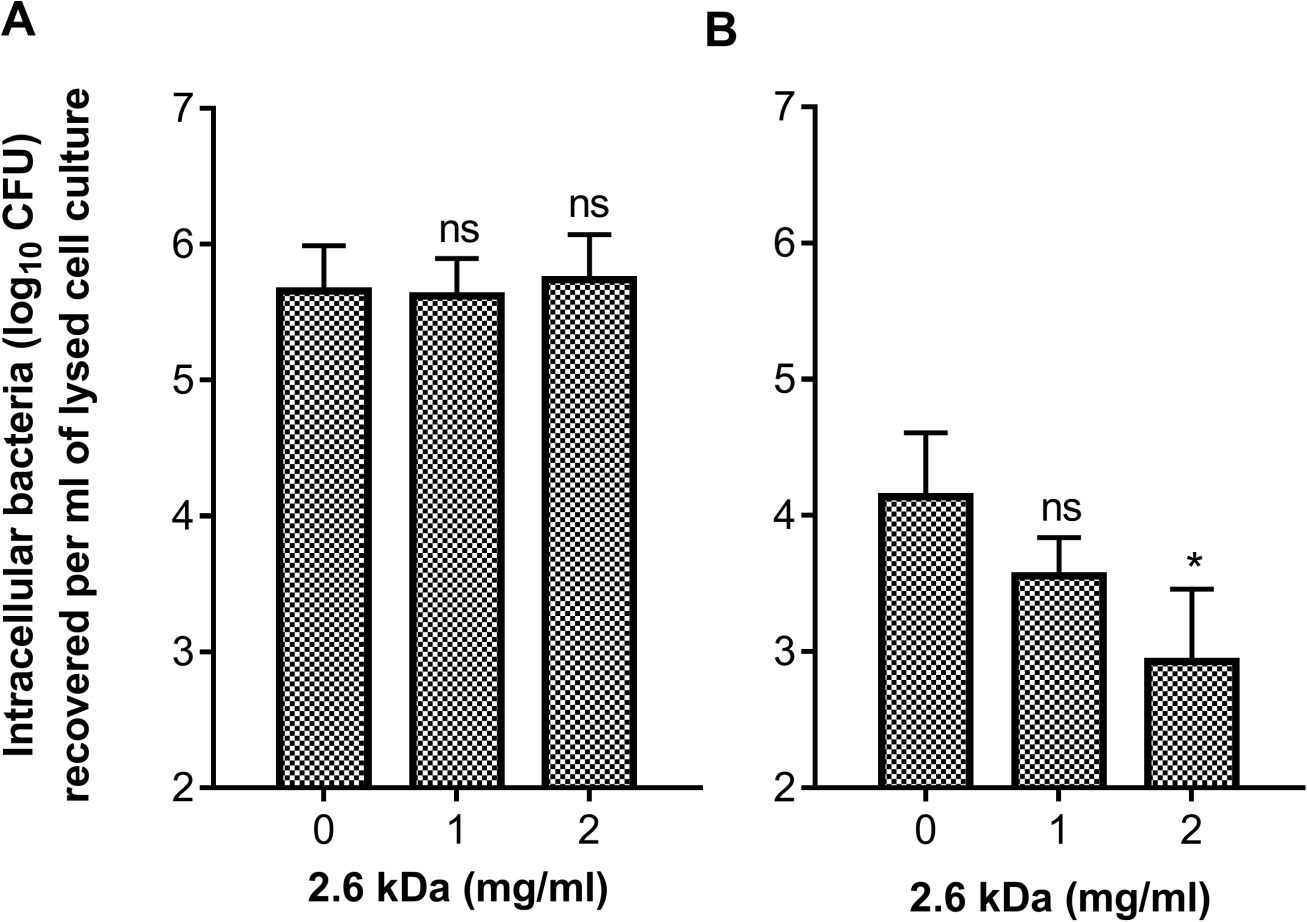
**Evaluation of internalization (A) and persistence (B) of the MRSA strain 1158c into MAC-T cells after exposure to the 2.6 kDa chitosan used at different concentrations**. In A, MAC-T cells were exposed to bacteria and chitosan for a period of 3h. Cell cultures were then supplemented by lysostaphin to lyse extracellular bacteria, which are then removed, together with chitosan, through washing before provoking MAC-T cell lysis for determination of the number of intracellular bacteria at 3 h. In B, instead of lysing the MAC-T cells at 3 h, the number of persisting intracellular bacteria was determined at 24 h. Data were obtained from three independent experiments. Significant differences in comparison to the untreated control (0 mg/ml) are shown. Statistical analysis was performed using one way ANOVA with Dunnett’s multiple comparison: *, *P*<0.05.

MAC-T bovine mammary epithelial cells were used to evaluate the ability of the 2.6 kDa chitosan to prevent the internalization and the intracellular persistence of MRSA strain 1158c. A 3-h exposure to chitosan does not prevent the internalization of ~6 log10 CFU/ml of the MRSA strain into MAC-T cells at the concentrations tested (MIC and 2 × MIC; Fig 8A). However, chitosan used at 2 mg/ml was able to significantly reduce the persistence of viable bacteria within the MAC-T cells after 24 h compared to the untreated control (Fig 8B). Note that chitosan was removed from the cell culture after allowing internalization of bacteria (*i*.*e*., after the 3-h incubation period in the presence of chitosan). Since the bactericidal effect of chitosan is seen after internalization of bacteria, this suggests that the 2.6 kDa chitosan was internalized together with bacteria during the 3-h incubation period.

### Efficacy of chitosan for treatment of *S*. *aureus* intramammary infection in the mouse

**Fig 9:**
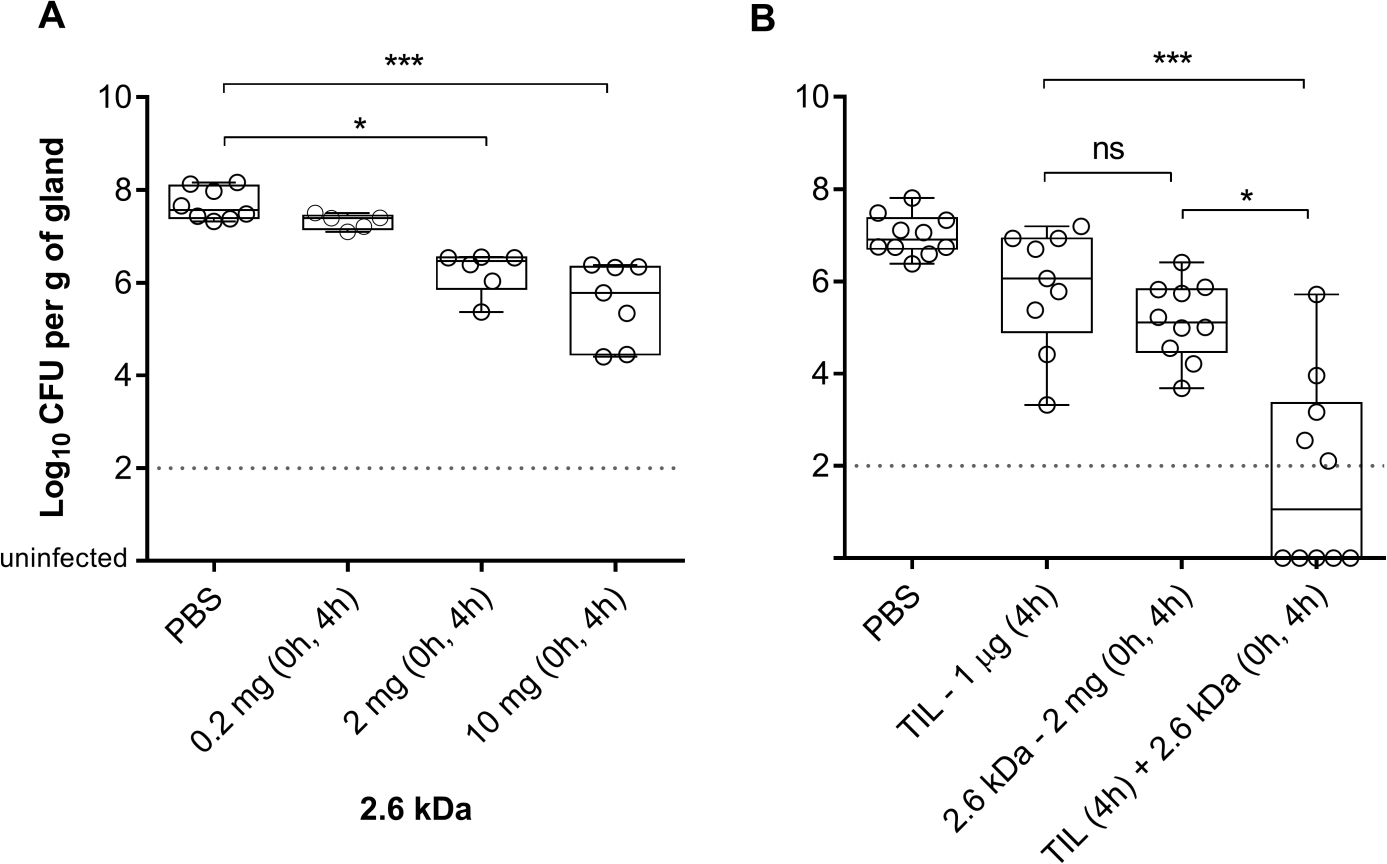
Efficacy of the 2.6 kDa chitosan used alone or in combination with tilmicosin on *S*. *aureus* in a mouse mastitis model. (A) Chitosan was administered twice at (0 h and 4 h post-inoculation) using 0.2, 2 or 10 mg/gland for the challenge against *S*. *aureus* strain Newbould. (B) A challenge with *S*. *aureus* strain 2117 was treated with the 2.6 kDa chitosan or tilmicosin (TIL) or a combination of both. Chitosan was administered twice at (0h and 4 h post-inoculation) whereas tilmicosin was administered 4 h post-inoculation. The middle bars indicate median values for each group of glands (n = 5–8) whereas the boxes specify quartiles Q1-Q3. The detection limit was 200 CFU (dotted line), below that, the glands were considered uninfected. Statistical analysis was performed using the Kruskal-Wallis test: *, *P*<0.05; ***, *P*<0.001.

We evaluated the capacity of chitosan alone or in combination with an antibiotic to reduce *S*. *aureus* colonization of mouse mammary glands. First, the antibacterial activity of the 2.6 kDa chitosan used alone was assessed against *S*. *aureus* strain Newbould (Fig 9A). Newbould is a reference bovine strain often used in this murine model [41, 42]. Results show that two intramammary instillations of either 2 mg or 10 mg of chitosan were able to significantly reduce colonization of the mammary glands by strain Newbould (Fig 9A).

Moreover, we tested the ability of chitosan to potentiate the activity of tilmicosin against the biofilm hyperproducer strain *S*. *aureus* 2117 (Fig 9B). In this test, two instillations of chitosan at 2 mg/gland were able to enhance the antibacterial activity of tilmicosin by significantly reducing the bacterial counts in glands as compared to the effect obtained with a treatment by tilmicosin or chitosan alone. This supports the antibacterial synergy found in the checkerboard and bactericidal assays performed *in vitro*.

## Discussion

Bovine mastitis is considered one of the most recurrent and costly disease of dairy cattle [42]. Mastitis usually affects milk production and quality by an increase of its somatic cell count [43]. Additionally, treatment of mastitis increases the risk of antibiotic residues in milk which alter its intrinsic properties [44]. Alternative therapies using molecules such as chitosan could avoid some of the problems associated with antibiotics. Chitosan is a biodegradable molecule that has been used as a drug carrier in some treatment applications [45]. Besides, the antimicrobial activity of chitosan itself has been reported in some studies against a wide range of bacteria [26]. The molecular weight (MW) or degree of polymerization (DP), and the degree of deacetylation (DD) are the main factors modulating chitosan biological activities [28]. However, a variety of forms and mixtures have been used and reported in the scientific literature rendering difficult the association of a specific form of chitosan to a specific biological activity. In this study, we used well defined forms of chitosan to adequately associate some forms to important biological activities against *S*. *aureus*, notably the antibiofilm and bactericidal activities of chitosan against this pathogen responsible for a large number of IMIs worldwide [46, 47, 48].

In the present study, susceptibility tests showed that all the forms of chitosan we have tested exhibited an antibacterial effect against *S*. *aureus*. The MICs of the 1.3, 2.6 and 4.0 kDa were however much lower than those obtained with CHOS (0.4–0.6 kDa). Hence, we showed here that the DP is critical for activity and that an oligosaccharide of at least 6 units provides maximum antibacterial activity against *S*. *aureus* when chitosan is highly deacetylated (DD of 98%). Many studies on the antibacterial activity of chitosan have indeed reported a correlation between its activity and its MW but the use of chitosans of diverse provenance may have generated conflicting results. Some studies described that high chitosan polymerization yielded low activity against *E*. *coli* [49], while larger chitosan forms showed greater activity than smaller chitosan molecules in another study [50]. One more study reported that a concentration of 1.2 mg/ml of chitosan exhibited a good inhibitory activity against *S*. *aureus* but the DP of those chitosan molecules was not characterized [51]. Besides, the mode of action of chitosan is still not well understood and it is possible that different forms of chitosan have different modes of action. The polycationic structure of chitosan is associated with its antimicrobial activity [52]. Transmission electron microscopy localized chitosan molecules on bacteria cell surfaces with “vacuole-like” structures underneath the wall [53]. Its action, leading to cell membrane lysis, has been also depicted in Gram negative and Gram positive bacteria [54]. Some studies have reported that chitosan interacts with the DNA of bacteria and fungi, thus preventing RNA and protein syntheses [55, 56, 57].

Chitosan can also exhibit a synergy with antibiotics. Using a checkerboard assay, we found that the 2.6 kDa chitosan exhibited a synergy with macrolides such as erythromycin and tilmicosin. Tilmicosin is approved for treatment of bovine infections and could thus be of interest as a potential drug partner for chitosan for the treatment of IMIs in dairy cattle. Other studies have reported a synergy of chitosan with disinfectants (chlorhexidine) or antibiotics (sulfamethoxazole) for combating dental plaque or improving antibiotic activity against *Pseudomonas aeruginosa*, respectively [58, 59].

As previously mentioned, biofilm production is a significant contributor of *S*. *aureus* pathogenesis. Therefore, we have also investigated the antibiofilm activity of the different forms of chitosan available in our lab. Like reported for *Candida albicans* [60], our chitosan molecules with the highest molecular weight had the most antibiofilm activity against *S*. *aureus*. This was evidenced using the biofilm/growth ratio to rule out direct growth inhibition. In fact, examining the effect of chitosan on preformed biofilms from strain MRSA 1158c and the biofilm-hyperproducer strain 2117, we clearly demonstrated that the antibiofilm activity of the 2.6 kDa chitosan affects biofilm formation at concentrations much below the MIC, at least for strain 2117.

Interestingly, some studies reported that the biofilm matrix composition differs between that produced by MRSA and that from methicillin-susceptible (MSSA) strains. Indeed, *S*. *aureus* is able to form biofilms by *ica*-dependent and *ica*-independent pathways. The *ica*-dependent biofilm formation in MSSA is mostly mediated by the polysaccharide intercellular adhesin or poly-N-acetyl-glucosamine (PIA/PNAG), which is produced by enzymes encoded by the *icaADBC* operon, whereas the *ica*-independent biofilm formation in MRSA is mediated by protein-protein interactions [61, 62]. This possible difference in the biofilm matrices of our test strains (MRSA 1158c *vs*. 2117) may perhaps explain why the biofilm of strain 2117 was much more affected by chitosan in our study. Many antibiofilm polysaccharides, including chitosan, are believed to modify the physical characteristics of bacterial cells and abiotic surfaces. However, some reports mentioned that polysaccharides may act as signaling molecules that modulate gene expression of recipient bacteria leading to biofilm disruption or inducing cell motility for dissemination of cells from biofilms [63, 64, 65]. It remains to be seen if the regulatory pathway by which strain 2117 hyperproduces biofilms compared to other *S*. *aureus* strains [19] can be specifically modulated by chitosan.

We also report here the bactericidal activity of the 2.6 kDa chitosan used alone or in combination with tilmicosin against bacteria embedded in preformed biofilms. Whether chitosan helps disrupting the biofilm thus helping the antibiotic to act on bacteria that are released from the biofilm or whether chitosan combines its own bactericidal action to the bacteriostatic effect of this macrolide antibiotic for a one-two punch against the embedded bacteria, is still unknown. It was recently proposed that chitosan improves gentamicin efficacy in biofilms by facilitating the penetration of this aminoglycoside into the biofilm architecture of *Listeria monocytogenes* [66]. This is indeed yet another possibility to explore.

The many interesting characteristics of chitosan such as biocompatibility, low toxicity and biodegradability allow its use in many applications [67]. In the present study, we have shown that the 2.6 kDa chitosan was non-cytotoxic for bovine mammary epithelial cells at a concentration of at least 2 × MIC and that this concentration significantly reduced the persistence of intracellular bacteria. We did not observe any effect of the 2.6 kDa chitosan on the internalization of *S*. *aureus* into MAC-T cells over a 3-h period. In contrast, it was reported that chito-oligosaccharides (CHOS, DP range of 2–20) at concentrations of 1–16 mg/ml were able to reduce the adherence of Gram negative bacteria onto eukaryotic cells [68], indicating that similar forms of chitosan may affect distinct microbial species differently. The anti-adherence effect of oligosaccharides has been assigned to the resemblance between the oligosaccharide structure and cell surface receptors to which some bacteria attach prior to colonization [68].

We showed here that among the chitosan forms tested, the 2.6 kDa molecule appeared innocuous in mice and in cows after intramammary instillation. This was in contrast to the 4.0 kDa molecule, which caused visible signs of inflammation. It was therefore sensible to use the 2.6 kDa chitosan as a therapeutic agent alone or in combination with tilmicosin in a *S*. *aureus* IMI model. We found that the 2.6 kDa chitosan was indeed able to significantly reduce the bacterial counts in mammary glands in a dose-response manner. In addition, the combination of the 2.6 kDa molecule and tilmicosin was significantly more effective than chitosan or tilmicosin used alone. Such an effect of the combination confirmed the bactericidal synergy observed against planktonic cells in susceptibility tests and against bacteria embedded in preformed biofilms *in vitro*.

Many factors may account for the observed efficacy of chitosan in the IMI model. The reduction of mammary gland colonization by *S*. *aureus* could indeed be explained in part by the bactericidal activity of chitosan molecules as observed *in vitro*. Similarly, the antibiofilm activity of chitosan observed *in vitro* may have also contributed to some extent, although this is rather difficult to demonstrate *in vivo*. In future studies, it would be interesting to see if particular formulations increase further the combined actions of chitosan and tilmicosin *in vivo*. Liposomes have been used to improve the stability and activity of a variety of antibiofilm molecules [69, 70, 71]. Preparation of liposomes from milk fat globule membrane phospholipids might represent an interesting avenue [72].

## Conclusion

This study highlighted the antibiofilm and bactericidal properties of the 2.6 kDa chitosan with a degree of polymerization of 13 and a high degree of deacetylation (98%) against MSSA and MRSA strains of bovine origin. The use of biodegradable and innocuous compounds such as chitosan alone or in combination with low concentrations of antibiotics like tilmicosin, may represent an alternative treatment for IMIs caused by *S*. *aureus* and could notably help reducing antibiotic use in dairy farms.
